# A Survey of Preclinical Studies Evaluating Nanoparticle-Based Vaccines Against Non-Viral Sexually Transmitted Infections

**DOI:** 10.3389/fphar.2021.768461

**Published:** 2021-11-24

**Authors:** Abisola Abisoye-Ogunniyan, Isabella M. Carrano, Dina R. Weilhammer, Sean F. Gilmore, Nicholas O. Fischer, Sukumar Pal, Luis M. de la Maza, Matthew A. Coleman, Amy Rasley

**Affiliations:** ^1^ Biosciences and Biotechnology Division, Lawrence Livermore National Laboratory, Livermore, CA, United States; ^2^ Department of Plant and Microbial Biology, Rausser College of Natural Resources, University of California, Berkeley, Berkeley, CA, United States; ^3^ Department of Pathology and Laboratory Medicine, University of California, Irvine, Irvine, CA, United States

**Keywords:** STIs, vaccines, nanoparticles, delivery platforms, immunogenicity, chlamydia, syphilis, gonorrhea

## Abstract

A worldwide estimate of over one million STIs are acquired daily and there is a desperate need for effective preventive as well as therapeutic measures to curtail this global health burden. Vaccines have been the most effective means for the control and potential eradication of infectious diseases; however, the development of vaccines against STIs has been a daunting task requiring extensive research for the development of safe and efficacious formulations. Nanoparticle-based vaccines represent a promising platform as they offer benefits such as targeted antigen presentation and delivery, co-localized antigen-adjuvant combinations for enhanced immunogenicity, and can be designed to be biologically inert. Here we discuss promising types of nanoparticles along with outcomes from nanoparticle-based vaccine preclinical studies against non-viral STIs including chlamydia, syphilis, gonorrhea, and recommendations for future nanoparticle-based vaccines against STIs.

## 1 Introduction

Sexually transmitted infections (STIs) are among the most common public health burdens worldwide (Thomas et al., 2019). They are the leading cause of severe reproductive health complications, congenital infections, and increased risk for acquiring other STIs such as human immunodeficiency virus (HIV) (Sexton et al., 2005; Barrow et al., 2020). STIs are caused by a range of bacteria, viruses, and parasites and are transmitted primarily *via* sexual contact (Wagenlehner et al., 2016; Dagnew et al., 2020). Many STI infections may present as asymptomatic diseases, which compounds the difficulty in overcoming associated disease burden (Wi et al., 2019). According to the World Health Organization (WHO), eight of these pathogens are responsible for the majority of STIs. Four of the eight infections are curable STIs, namely chlamydia, syphilis, gonorrhea, and trichomoniasis. The other four infections are incurable viral infections, including HIV, hepatitis B, human papillomavirus (HPV), and herpes simplex virus (HSV) (World Health Organization, 2018). Globally, over 1 million STIs are acquired daily (World Health Organization, 2018), and the high incidence of infected persons presenting with complications and other sequelae calls for extensive research on the development of new preventive measures.

Vaccines have been the most effective means for the control as well as potential eradication of infectious diseases (Reljic and González-Fernández, 2019). Over the years, global efforts focused on lowering vaccine-preventable diseases has led to tremendous advancements in vaccine development against infections including STIs. Traditional vaccines such as live-attenuated whole-pathogen, inactivated whole-pathogen, subunit, and nucleic acid vaccines have been studied for their potential to safely induce protective immune responses. While not every infectious agent can be effectively targeted with whole-pathogen or subunit vaccines, those that can be targeted have been met with undesired side effects. These effects include reversion to virulence of live-attenuated whole pathogens, development of more severe infection outcomes following vaccination with inactivated whole pathogens (Rose et al., 2018), as well as the need for routine booster shots to attain robust protection against diseases (Vartak and Sucheck, 2016). Vaccine development against STIs has faced additional challenges, ranging from socio-cultural vaccine acceptance to the selection of appropriate antigens, availability of adjuvants, and delivery systems suitable for humans (Zimet et al., 2000; Gottlieb and Johnston, 2017; Reljic and González-Fernández, 2019).

Subunit vaccines, in which only components of infectious agents are used instead of the entire pathogen, have shown significant promise for the development of safer vaccines against STIs. However, studies have shown that administering the subunit antigens alone is not sufficient to produce adequate long-term immunity needed to prevent disease. Major obstacles to vaccine development include difficulty in the identification and production of appropriate antigens for biological specificity (Flower et al., 2010; Vartak and Sucheck, 2016), identification of suitable adjuvants that will induce a robust protective immune response when formulated with antigens of interest, and the optimization of antigen-adjuvant interactions for maximum vaccine immunogenicity and stability (Fox et al., 2013). To address the need for suitable adjuvants, as well as effective and safe delivery platforms for promising antigens, researchers have adopted the use of nanoparticles as they provide facile platforms that can be designed to specifically improve immunogenicity with high efficacy (Pati et al., 2018).

In this article, we review the implications of nanoparticle-based vaccines and the outcomes of current delivery systems used in nanoparticle-based vaccines against non-viral pathogens, including the bacterial pathogens *Chlamydia trachomatis* (chlamydia), *Treponema pallidum* (syphilis), and *Neisseria gonorrhoeae* (gonorrhea). Given the success of nanocarriers used in the current COVID-19 vaccines and the early data from the CTH522 Chlamydia vaccine clinical trials (NCT02787109 and NCT03926728), we envisage that nanocarriers will continue to emerge as important components of pre-clinical vaccine studies especially for bacterial pathogens to dramatically change the current status of vaccines against STIs. In many ways, the preclinical vaccine studies discussed in this review are aspirational, however, we believe the timing is right to explore these concepts in the context of vaccines for bacterial STIs. We conclude with recommendations for future improvements in the efficacy and application of nanoparticle-based vaccines against STIs.

## 2 Implications of Nanotechnology in Vaccine Development for Sexually Transmitted Infections

For the past several decades, there has been increasing application of nanotechnology in fields including biology and the biomedical sciences, materials science, chemistry, and physics (Wang and Wang, 2014; Bayda et al., 2019). Nanoparticles are small particles that typically range between 1 and 100 nm in size and display disparate physical and chemical properties when compared to their larger bulk material equivalents (Jeevanandam et al., 2018), as their high surface area to volume ratio increases reactivity at the molecular level (Mourdikoudis et al., 2018). This intrinsic feature of nanoparticles allows them to exhibit distinct physical, chemical, and optical properties that enhance their versatility (Mourdikoudis et al., 2018) to be engineered for enhanced immune modulation and tailored antigen delivery.

**FIGURE 1:**
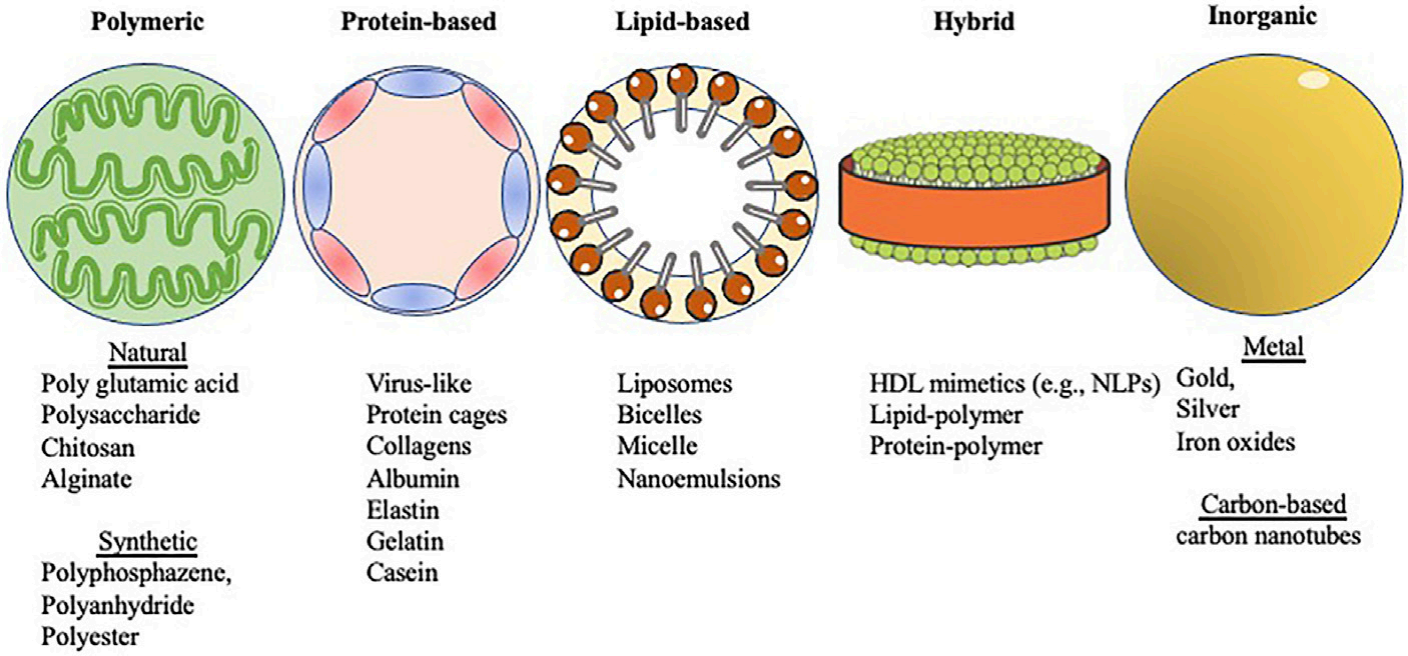
Shown in abstract form are five different types of nanoparticles covered within this review. The categories were selected based on the major constituents that form the different nanoparticles (see Section 3 text). Images were adapted from Servier Medical Art by Servier, which is licensed under a Creative Commons Attribution 3.0 Unported License. Different forms of nanoparticle scaffolds for vaccine development.

Nanoparticle-based vaccines take advantage of a combination of nanoparticle size and composition. Their small size provides high surface area relative to bulk materials and mimics the size of particulates against which our immune systems are primed (e.g., viruses) (Pati et al., 2018). The disparate compositions of nanoparticles provide a diverse toolkit for vaccine formulation and enables fine-tuning of key physicochemical properties such as charge, functionalization, and stability (Chattopadhyay et al., 2017). These characteristics (Table 1), in turn, can be used to tune the biological effects of nanoparticle-based vaccines, including delivery, cellular uptake, biodistribution, stability, antigenicity, and efficacy (Al-Halifa et al., 2019). Nanoparticle-based vaccines are engineered from a variety of biodegradable materials including lipids, proteins, natural and synthetic polymers, as well as inorganic materials that exhibit important antigenic moieties (Pati et al., 2018) (Figure 1). Nanotechnology in vaccine development for STIs also accelerates the possibility for targeted antigen delivery, and regulated discharge of antigens to antigen presenting cells (APC)s (Peek et al., 2008). The conjugation or encapsulation of antigens to nanoparticles provides protection from proteolytic degradation while improving and prolonging antigen delivery to immune cells, a requirement that will facilitate the robust protection needed against STIs (Gao et al., 2018; Pati et al., 2018; Sahu et al., 2018). Various combinations of antigens and adjuvants can be prepared using nanoparticle platforms to generate both humoral immune responses and cellular immunity against infections (Mohan et al., 2013; Dykman, 2020), as well as generate longer lasting immunity, which is necessary for simplifying vaccination schedules (Lofano et al., 2020).

**TABLE 1:** Size, constituents and benefits of key nanocarriers.

Nanoparticle platform	Nanocarrier	Size range	Constituents	Benefits	Refs
Polymeric Nanoparticles	Poly-γ-glutamic acids	150–250 nm	Polyamino acid formed by the amide bond linkage between the amino group on the α-carbon and the carboxyl group on the γ-carbon	Naturally occurring anionic homopolyamide	Akagi et al. (2005); Kim et al. (2019)
				Biodegradable and biocompatible	
				Good water solubility	
				Nontoxic and edible	
				Non-immunogenic	
	Polysaccharides	Varying between 10 and 850 nm depending on polysaccharide backbone	Chitosan: β-(1–4)-linked d-glucosamine and N-acetyl-d-glucosamine	Biodegradable and biocompatible	Arora et al. (2016); Gericke et al. (2020)
			Starch: branched amylopectin and linear amylose	Easy surface modification	
			Alginate: two sterically dissimilar repeating units 1,4α-l-gluconate and 1,4β-d-mannuronate	Nontoxic and non-immunogenic	
			Dextran: 1,6-linked d-glucopyranose units	Enhanced drug delivery	
			Pullulan: maltotriose units (α-1,4-; α-1,6-glucan)	Long shelf life	
				Natural origin and easily available	
				Efficient encapsulation of a wide range of proteins as well as hydrophobic and hydrophilic compounds	
				Capability to be tuned to release encapsulated antigens or drugs for a desired duration	
	Polyphosphazene	150–700 nm	Carboxylic acid and pyrrolidone moieties attached to inorganic phosphorus-nitrogen backbone	Highly biodegradable	Martinez et al. (2017)
				Structural diversity	
				Protein binding ability and endosomolytic	
				Environmentally triggered self-assembly into nanoparticulate carriers	
	Polyanhydride	250 nm–3 μm	1,6-bis(p-carboxyphenoxy) hexane, 1,8-bis(p-carboxyphenoxy)-3,6-dioxaoctane, and sebacic acid	Biocompatibile	Kelly et al. (2020); Norris et al. (2020); Thukral et al. (2020)
				Exhibit adjuvant-like properties	
				Highly internalized by APCs	
				Ability to both humoral and cell-mediated immune responses	
				Ability to stabilize encapsulated payloads	
				Superior retention of protein stability	
				Erosion-controlled sustained release	
Protein-Based Nanoparticles	Viruses	10–100 nm	Protein building blocks	Highly stable	Koudelka et al. (2015); Bhaskar and Lim, (2017)
				High biocompatibility and biodegradable	
				Efficient delivery of cargo to target cells	
				Naturally immunogenic	
				Ability to cross biological barriers	
	Protein cages	Few nanometers up to ∼500 nm	Varying compositions with three distinct interfaces: interior surface, exterior surface and interfaces between subunits	Highly biocompatible	Aumiller et al. (2018); Choi et al. (2018)
				Well-defined architectures	
				Effective drug delivery protein	
	Collagens	Hundreds of nm in diameter with 67 nm repeating bank structures	Amino acid residues	High biocompatibility and biodegradable	Nagarajan et al. (2014); Wang et al. (2014); DeFrates et al. (2018)
				Low antigenicity	
				Advantageous for some administration routes including pulmonary and oral delivery	
				Capable of reassembling the microenvironment allowing for effectively delivery of drugs	
	Albumin	50–300 nm	Amino acid residues	Easy to prepare and reproducible	Elzoghby et al., (2012); Karimi et al. (2016)
				Well tolerated	
				Nontoxic, non-immunogenic, biocompatible, and biodegradable	
				Its structural flexibility allows reversible binding	
				Binds naturally to hydrophobic molecules with non-covalent reversible binding	
				Easily carry hydrophobic molecules into the bloodstream via endogenous albumin pathways	
	Elastin	300–400 nm	A repeating sequence of pentapeptides, (Val-Pro-Gly-X-Gly) n where X can be any amino acids except for proline	Biocompatible	Lohcharoenkal et al. (2014); Monfort and Koria. (2017)
				Highly soluble	
				Tunable transition temperature	
				Genetically encodable and immunogenic	
	Gelatin	200–500 nm	A poly-ampholyte consisting of both cationic and anionic groups	Nontoxic and inexpensive	Lee et al. (2011); Azimi et al. (2014)
				Easily available and bioactive	
				Biodegradable and biocompatible	
				Great thermal range	
				Presence of abundant active groups	
				Effective drug delivery protein	
	Casein	50–500 nm	α_s1_-, α_s2_-, β-, and κ-caseins.	Nontoxic and highly stable	Malekhosseini et al. (2019)
				High encapsulation efficiencies	
				Suitable carriers for different nutraceuticals	
				Bioavailable and an important source of essential amino acids, phosphate, and calcium	
	Liposomes	50–450 nm	Phospholipids: amphiphilic molecules with a hydrophilic or charged head and two nonpolar hydrophobic chains	Biocompatible and biodegradable	Wang and Wang, (2014); Bozzuto and Molinari. (2015); Lujan et al. (2019)
				Diverse range of composition	
				Efficient encapsulation of biomolecules	
	Bicelles (bilayered discoidal micelles)	20–50 nm	Lipid bilayer stabilized by detergent molecules	Tunable size	Dürr et al. (2012); Geisler et al. (2019); Shen et al. (2019)
				Increased internalization by tumor cells. compared to liposomes	
				Increased ability to penetrate through tissues	
	Micelles	5–100 nm	Amphiphilic surfactant molecules with a hydrophilic head and a hydrophobic tail, typically long hydrocarbon chains	Efficient encapsulation of hydrophobic drugs to increase solubility	Husseini and Pitt, (2008); Tyrrell et al. (2010)
				Increased bioavailability of drugs	
				Stabilization of nanoemulsions	
	Nanoemulsions	10–1,000 nm	Hydrophobic liquid core stabilized by surfactant	Increased bioavailability of drugs	Husseini and Pitt. (2008); Jaiswal et al. (2015)
				Prolonged drug delivery	
				Increased solubilization of lipophilic drugs	
				Non-toxic and non-irritant	
				Great substitute for liposomes	
				Enhances absorption due to their small-sized droplets with increased surface area	
Hybrid Nanoparticles	Phospholipid bilayer shell	205–295 nm	Poly (β-amino ester) poly-1 (or PLGA for pH-insensitive control particles)	Low toxicity	Su et al. (2011)
			Phospholipids DOPC, DOTAP, and DSPE-PEG in a 7:2:1 M ratio	Efficient encapsulation of the polycation core	
			Dicholoromethane (DCM)	Efficient adsorption of mRNA.	
	Lipid-polymer hybrid nanoparticle	10–500 nm	PLGA	High colloidal stability	Rose et al. (2015)
			DDA	Prolonged antigen and/or immunopotentiator delivery	
			Trehalose-6,6′-dibehenate (TDB)- CAF01		
	Telodendrimer NLP	∼40 nm	mMOMP DNA	E. coli-based cell-free expression system	He et al. (2017)
			Δ49apolipoprotein A1 (Δ49ApoA1) DNA	High-yield production (model for difficult-to-obtain antigens	
			Lipids and 1,2-dimyristoyl-sn-glycero-3-phosphorylcholine (DMPC)/Telodendrimer PEG5000-CA8 nanolipoprotein particle	Non-toxic	
			CpG	Great substitute for liposomes	
				Synthesis is very flexible	
				Stable with low aggregation	
	NLP bilayer	∼40 nm	Phospholipids 1,2-dioleoyl-sn-glycero-3-phosphocholine (DOPC) synthetic monophosphoryl Lipid A (MPLA)	Highly stable	Weilhammer et al. (2017); Weilhammer et al. (2020)
			N-Hydroxysuccinimide-polyethyleneglycol 4-dibenzylcyclooctyne (NHS-PEG4-DBCO)	Effective colocalization of adjuvant and antigen	
			NLP scaffold protein apoE422k/nickel-chelating NLPs (NiNLPs)	Suitable for subunit vaccine delivery	
			Model antigen ovalbumin (OVA)/anthracis antigen	Well tolerated	
				Permits multiple routes of delivery	
Inorganic Nanoparticle	Gold	2–100 nm (15–50 most effective)	Colloidal gold particles	Low toxicity	Dykman and Khlebtsov (2012); Dykman. (2020)
				Inherent adjuvant properties	
				Geometrically manipulatable	
	Silver	1–100 nm	Metallic colloidal silver particles	Antimicrobial and bactericidal	Iravani et al. (2014)
				High surface area to volume ratio	
	Iron oxides	10–100 nm	Synthetic γ-Fe2O3(maghemite) or Fe3O4 (magnetite) particles with an organic or inorganic coating	Sufficient biocompatibility	Wahajuddin and Arora. (2012)
				Physical and chemical stability	
				Increased targeting capability using supermagnets	
	Carbon nanotubes	Single-walled carbon nanotubes: 0.7 and 3 nm	Single-walled carbon nanotubes: single layer of graphene sheet.	Highly stable, non-immunogenic with low toxicity	Bianco et al. (2005); Donaldson et al. (2006)
		Multi-walled carbon nanotubes 10–200 nm	Multi-walled carbon nanotubes: many layers of graphene sheet to form concentric cylinders	Large surface area	
				Efficient conjugation of multiple antigens simultaneously	
				Biocompatible	
				High propensity to cross cell membranes	
				Can be charged with biologically active moieties	

Modern vaccine design has benefited from the use of nanoparticles, as seen in recent times with the nanoparticle-based vaccines targeting SARS-CoV-2 (NCT04368728) (Shin et al., 2020; Walls et al., 2020). In their preclinical study, the authors described a very effective nanoparticle-based vaccine engineered with a self-assembling protein nanoparticle displaying 60 SARS-CoV-2 spike receptor-binding domains in a highly immunogenic array and was shown to induce a 10-fold higher neutralizing antibody titer even at a 5-fold lower dose than the prefusion-stabilized spike ectodomain S-2P trimer (Walls et al., 2020). The overall benefits of nanoparticle-based vaccines in general vaccine applications cannot be overemphasized (Peek et al., 2008; Nasir, 2009). Hence, the use of nanoparticle-based vaccines as a substitute to the traditional vaccines offer a very interesting and promising approach for vaccine delivery against STIs.

## 3 Nanoparticle-Based Vaccine Delivery Platforms

The different types of biodegradable materials from which nanoparticles are engineered offer a wide variety of delivery platforms for vaccines, such as protein-based nanoparticles, natural and synthetic polymeric nanoparticles, lipid-based nanoparticles, and hybrid nanoparticles (Al-Halifa et al., 2019). These biodegradable nanomaterials are usually preferred over inorganic nanoparticles due to the latter’s propensity for particle aggregation (Zhu et al., 2014; Al-Halifa et al., 2019) (Table 2).

**TABLE 2:** *In Vivo* applications of nanoparticle-based vaccines against STIs.

STIs	Nanoparticle platform	Nanocarrier	Antigen	References
Chlamydia	Biodegradable polymericnanoparticle	Chitosan	MOMP	Cambridge et al. (2013)
	Lipid-polymer hybrid nanoparticle	Telodendrimer NLP	Chlamydia muridarum MOMP protein	He et al. (2017)
	Lipid-polymer hybrid nanoparticle	PLGA	Chlamydia trachomatis recombinant MOMP protein	Rose et al. (2015)
	Lipid-polymer hybrid nanoparticle	Glycol-chitosan-coated lipid-polymeric hybrid nanoparticle	Recombinant fusion antigen CTH522	Rose et al. (2018)
	Biodegradable polymeric nanoparticle	PLGA (85:15)	Chlamydia trachomatis recombinant MOMP protein	Sahu et al. (2020)
	Biodegradable polymeric nanoparticle	PLGA (50:50)	Chlamydia trachomatis recombinant MOMP-187 peptide	Taha et al. (2012)
	Biodegradable polymericnanoparticle	PLGA (50:50)	Chlamydia trachomatis recombinant MOMP protein	Fairley et al. (2013)
	Biodegradable polymericnanoparticle	PLA-PEG	Chlamydia M278 MOMP	Dixit et al. (2018); Verma et al. (2018)
	Biodegradable polymeri nanoparticle	PLA-PEG	Chlamydia trachomatis recombinant MOMP protein	Dixit et al. (2014)
	Protein-based nanoparticle	Vault- A natural nanocapsule made from hollow barrel shaped eukaryotic ribonucleoprotein complexes	Polymorphic membrane protein G-1 (PmpG) peptide	Jiang et al. (2017)
	Self-assembling, Lipid-based nanoparticle	Lipid phytantriol (Phy) and monomycoloyl glycerol-1 (MMG-1)	Chlamydia trachomatis MOMP	Rodrigues et al. (2018)
Syphilis	Biodegradable polymeric nanoparticle	Chitosan	Tp92	Zhao et al. (2011)
	Biodegradable polymeric nanoparticle	Chitosan	Gpd	Zhao et al. (2013)
Gonorrhea	Protein-polymer hybrid nanoparticle	Crosslinked albumin polymer matrix microparticles	Spray dried, inactivated whole-cell gonococci strain CDC-F62	Gala et al. (2018)

### 3.1 Polymeric Nanoparticles

Many polymers are engineered as nanoparticle-based vaccine delivery platforms because of their biodegradability, biocompatibility, durability, and low toxicity. Polymers are extremely modifiable, hence their physiochemical properties can be readily modified to enhance their immunomodulatory effects (Bose et al., 2019). Polymeric nanoparticles enhance prolonged antigen delivery due to their increased stability *in vivo* (Thorp et al., 2020). This category can be further divided into two groups: natural and synthetic polymeric nanoparticles. Natural polymeric nanoparticles are further divided into poly glutamic acid-based nanoparticles and polysaccharide-based nanoparticles including chitosan, starch, alginate, and cellulose (Han et al., 2018). Chitosan-based nanoparticle vaccines, alginate-based nanoparticle vaccines, and their derivatives are the most studied polymeric materials as delivery platforms for vaccines against STIs, as well as other infectious diseases (Han et al., 2018; Bose et al., 2019). Many of these preclinical studies have shown the immunomodulatory effects of such nanoparticle-based vaccines against STIs. For example, several chitosan-based nanoparticle vaccines and therapies known for their intrinsic bio-adhesive properties have been shown to prolong interaction and contact duration with immune cells, which enhances their immunomodulatory effects (Prego et al., 2010; Zhao et al., 2013; Pradines et al., 2015; Rose et al., 2018). Synthetic polymeric nanoparticles include polyphosphazene, poly (glycolic acid) (PGA), poly (lactic acid) (PLA) and poly (DL-lactide-co-glycolic) acid (PLGA), poly (allymines)s, cell-penetrating poly (disulfide)s, and copolymer-based nanoformulations (Bose et al., 2019). Of all the synthetic polymeric nanoparticles, PLGA, a copolymer synthesized by the random polymerization of PLA and PGA, has been the extensively researched for vaccine delivery applications (Aikins et al., 2017).

### 3.2 Protein-Based Nanoparticles

Protein‐based nanoparticles are highly biodegradable and possess other properties including biocompatibility, biofunctionality, the ability to interact with other molecules for recognition, and their adaptive potential for genetic engineering for specific cell targeting and targeted protein delivery. They are known to exit the vasculature, enter the lymphatic system and permeate tissues through passive diffusion, making them highly beneficial as antigen delivery platforms. Protein-based nanoparticles are made by self‐assembly and desolvation. Examples of proteins that can be used for the fabrication of nanoparticles include collagen, albumin, elastin, gelatin, casein, whey proteins, silk proteins, soy proteins, and lectins (Young Kim et al., 2015; Qin et al., 2019). Some structurally complex and defined protein‐based nanoparticles include virus-like nanoparticles (VLPs), protein cages, and protein-based complexes (Diaz et al., 2018; Nguyen and Tolia, 2021). VLPs are derived from plant, microbial, insect, and mammalian viruses, without the viral genome itself, and are easily internalized by cells (Zeltins, 2013). VLPs are formed from the autonomous oligomerization or self-assembly of monomeric protein building blocks. These protein building blocks assemble into larger, stable and highly ordered structures that can be engineered to have a diameter ranging from 20 to 100 nm and possess unique physical properties (López-Sagaseta et al., 2016). Protein cages on the other hand include viral capsids, ferritins, and heat shock proteins, while protein-based complexes include Cry3Aa fusion protein platform (well known for the delivery of reporter proteins such as GFP in mammalian cells), silk proteins, and bovine serum albumin (Nair et al., 2015; Qin et al., 2019).

In cancer vaccine studies, peptide and protein-based vaccines typically fail to induce efficient immune responses against tumors. However, delivery of the peptides and proteins encapsulated in protein nanoparticles has shown promising improvements in vaccine efficacy characterized by increased immunogenicity of the tumor microenvironment. The highly organized structures and symmetry of protein nanoparticles as well as their ability to have specific functions inside or outside or in between subunits of the protein cage allows for the co-delivery of adjuvants and antigens that promotes antigen-specific immune responses against tumors (Neek et al., 2019). A ferritin nanoparticle vaccine was investigated for its ability to present *N. gonorrhoeae* peptide antigens on its surface. The *N. gonorrhoeae* peptides were inserted at the N terminus or in a surface-exposed ferritin loop between helices αA and αB. While crystal structures of the chimeric proteins from this study showed that the proteins were assembled correctly into a 24-mer nanocage structure, the inserted *N. gonorrhoeae* peptides were disordered on the nanocage surface showing multiple conformations (Wang et al., 2017). Protein nanoparticles have been utilized in many preclinical vaccine studies for hepatitis B, HPV and HIV with promising outcomes including the induction of antigen specific antibodies and viral clearance in animal models (Kirnbauer et al., 1992; Oh et al., 2004; Slupetzky et al., 2007; Paz De la Rosa et al., 2009; Tyler et al., 2014; Sliepen et al., 2015; Motevalli et al., 2018; Tokatlian et al., 2019; Wang et al., 2020; Ximba et al., 2020). These studies confirm the need for more extensive research to harness the potential of protein nanoparticles for vaccines against bacterial STIs that will allow for effective delivery of antigenic peptides and adjuvants.

### 3.3 Lipid-Based Nanoparticles

Most lipid‐based nanoparticles are derived from dietary oils or fats and are sometimes referred to as solid lipid nanoparticles based on their inclusion of lipids that exist as a solid phase at physiological temperatures. They include nanoemulsions, micelles, bicelles (bilayered discoidal micelles) and liposomes made from natural, semi‐synthetic or synthetic lipids, such as fatty acids, fatty alcohols, phospholipids, and medium and long chain mono-, di- and triglycerides. Lipid‐based nanoparticles are regarded as promising delivery platforms due to their ability to bypass multiple biological barriers, low toxicity, biocompatibility, and biodegradability (Niu et al., 2016; Qin et al., 2019).

Lipid-based nanoparticles have been used for the delivery of antigens including nucleic acids and proteins of infectious agents as well as cancer therapeutic agents to induce humoral immune responses (Aldosari et al., 2021; Lee et al., 2021; Park et al., 2021). These lipid-based nanocarriers pass through the cell membrane for cellular uptake by macropinocytosis, a type of endocytosis where they must escape from the endosomal lumen to deliver their enclosed antigens into the cytosol (Guevara et al., 2020; Lee et al., 2021). While ionizable lipid p*K*_a_ was thought to be the main factor involved in driving lipid nanoparticle potency and tolerability due to its effects on opsonization of the particles, cellular uptake and endosomal escape efficiency, it has been shown that other factors play a role in the immunogenicity of lipid nanoparticles (Hassett et al., 2019). Hence, a better understanding of mechanisms that regulate the biodistribution of antigen-loaded lipid nanoparticles will guide research outcomes for optimizing lipid nanoparticles as delivery systems.

### 3.4 Hybrid Nanoparticles

Hybrid nanoparticles incorporate key constituents from at least two different nanoparticle classes, leveraging the beneficial attributes of each to enhance their functionality. Hybrid nanoparticles include lipid-polymer hybrid nanoparticles, protein-dendrimer hybrid nanoparticles and high-density lipoprotein (HDL) mimetics (Qin et al., 2019). Lipid–polymer hybrid nanoparticles are core–shell nanoparticle structures made of polymer cores and lipid/lipid–PEG shells that show complementary properties in their biocompatibility and physical stability from both polymeric nanoparticles and liposomes (Hadinoto et al., 2013). Lipid–polymer hybrid nanoparticles exhibit higher *in vivo* cellular delivery when compared to either polymeric nanoparticles or liposomes. The formulation of lipid–polymer hybrid nanoparticles with chitosan has been shown to significantly increase mucosal immune responses against some STIs (Hadinoto et al., 2013; Rose et al., 2018).

In recent years newly engineered nanoparticles known as nanolipoproteins (NLPs), which are HDL mimetics consisting of discoidal lipid bilayer stabilized by apolipoproteins have been in development with desirable attributes for vaccine delivery. NLPs also known as nanodiscs are effective antigen carriers and supporting adjuvants that improve vaccine performance against infectious diseases. We have previously demonstrated that the use of NLPs enhances immune responses to both antigen and adjuvants *in vivo* (Fischer et al., 2013; Weilhammer et al., 2013; Weilhammer et al., 2017; Weilhammer et al., 2020). We have demonstrated that NLPs provide a membrane-like environment, which facilitate the embedding of functional membrane-bound proteins in their native conformations (He et al., 2017; Tifrea et al., 2021). The versatility of this type of nanoparticle underscores its potential for vaccines targeting bacterial and viral STIs.

### 3.5 Inorganic Nanoparticles

Inorganic nanoparticles include silica nanoparticles, metal nanoparticles (gold, silver, magnetic, metal organic framework-based nanoparticles), carbon-based nanomaterials (carbon nanotubes, nano-diamonds), and superparamagnetic nanoparticles (iron oxide nanoparticles). These nanoparticles are usually coated with organic molecules to make them biologically suitable as well as prevent particle aggregation. Commonly used inorganic nanoparticles for vaccine formulations include silver and gold nanoparticles. Gold nanoparticles are easily internalized by APCs and can facilitate their activation and elicit downstream immune responses, making them suitable platforms for antigen delivery (Bastús et al., 2009; Wang et al., 2018; Al-Halifa et al., 2019). A wide range of antigens and adjuvants can be conjugated on gold nanoparticles at high densities to prolong antigen delivery, improve immunogenicity as well as antigen presentation (Marques Neto et al., 2017). Although gold nanoparticles are a great platform for antigen conjugation, their ability to accumulate in organs such as the liver for long periods could ultimately result in toxicity (Arvizo et al., 2010). Coating with biocompatible materials has been shown to reduce their toxicity, although there is risk of alterations to their physicochemical and biological properties (Zhu et al., 2014).

## 4 Nanoparticle-Based Vaccines Against Sexually Transmitted Infections

Global estimates indicate that over one million STIs are acquired daily, suggesting a desperate need for the development of safe and efficacious vaccine formulations (Gottlieb and Johnston, 2017). We will highlight some of the preclinical studies dedicated towards nanoparticle-based vaccine research against chlamydia, syphilis, and gonorrhea in this section of the review and in Table 2.

### 4.1 Chlamydia

*C. trachomatis* is significant to human health worldwide (Paavonen and Eggert-Kruse, 1999; Dean, 2009; World Health Organization, 2018) as it remains the most diagnosed STI in developed countries despite the availability of effective and affordable therapy. Chlamydia exists either as an extracellular elementary body (EB), which is the non-replicating, infectious form or as a reticulate body (RB), the noninfectious form that replicates intracellularly (Nans et al., 2015; Elwell et al., 2016; Fischer and Rudel, 2018). The infection is mostly asymptomatic but can induce inflammatory responses at the site of infection, causing immunopathological sequelae such as pelvic inflammatory disease (PID), ectopic pregnancy, tubal infertility, miscarriage, and trachoma (an ocular disease) (Stamm, 1999; Vasilevsky et al., 2016; Lijek et al., 2018; Murthy et al., 2018). This has led to over 70 years of research dedicated to the development of vaccines that will provide adequate protection against *C. trachomatis* infection (Phillips et al., 2019). Chlamydial vaccines have utilized numerous approaches to improve vaccine immunogenicity, including the use of plasmid DNA, recombinant proteins, and subunit antigenic determinants, with or without adjuvants, yet the need for effective preventive measures remains unmet (Brunham and Rey-Ladino, 2005; Vasilevsky et al., 2014). Studies have indicated a critical role for chlamydia-specific CD4^+^ Th1 T cell responses in the clearing of infection and provision of protective immunity against chlamydia reinfection (Su and Caldwell, 1995; Gondek et al., 2012; Bakshi et al., 2018; Labuda and McSorley, 2018). The chlamydial major outer membrane protein (MOMP) has been shown to induce an immune response comparable to that seen with live bacteria in mice (Pal et al., 2005), while polymorphic membrane proteins (PMPs), have been explored for their ability to induce cross-species immunogenicity (Pal et al., 2017). MOMP and PMPs have been investigated as vaccine antigens in mice and nonhuman primates. Both antigens have shown suboptimal protection in many studies (Kari et al., 2009; Vasilevsky et al., 2016), hence the search for effective antigen-adjuvant combinations that can induce broad and long-lived protective immune responses continues (de la Maza et al., 2017).

The use of nanotechnology in the development of *C. trachomatis* vaccines is a promising strategy to enhance the production of long-lived immune protection. Preclinical studies exploring nanoparticle-based vaccines have shown effective delivery of immunogens and the induction of chlamydia-specific immune responses that will be beneficial in translational medicine. Chitosan nanoparticles possess the required size necessary to facilitate uptake by APCs for the induction of immune responses. They are shown to have high encapsulation capacity for stability and antigen protection from enzymatic digestion, making them favorable delivery platforms for chlamydial nucleic acids such as MOMP DNA constructs (Cambridge et al., 2013). Recombinant *C. trachomatis* fusion antigen CTH522 adjuvanted with glycol-chitosan-coated lipid-polymeric hybrid nanoparticle was shown to induce antigen-specific mucosal immune responses in the lungs and genital tract of mice, characterized by CTH522-specific IgG/IgA antibodies and IFN-γ-producing Th1 cells after nasal immunization (Rose et al., 2018).

PLGA nanoparticles have been extensively explored as vaccine delivery platforms due to their self-adjuvanting properties whereby they possess both antigenic and an adjuvanting moieties, their ability to prolong the release of antigens to stimulate dendritic cells as well as enhance intracellular antigen delivery to activated T-cells *via* the CD40 co-stimulation mechanism (Makadia and Siegel, 2011; Sneh-Edri et al., 2011; Rosalia et al., 2015). The lactide/glycolide ratio of PLGA nanoparticles is critical for efficient encapsulation and prolonged delivery of both antigens or drugs (Sahu et al., 2020), and PLGA nanoparticles at an 85:15 ratio used for the encapsulation of recombinant MOMP (PLGA-rMOMP) enhanced the activation of dendritic cells, induced chlamydia-specific rMOMP CD4^+^ memory and effector T-cells and activated an antibody adaptive immune response in immunized mice. Mice immunized intranasally with PLGA-rMOMP generated enhanced numbers of CD4^+^ T-cells but not memory and effector T-cells, whereas subcutaneously immunized mice produced both CD4^+^ memory and effector T-cells (Sahu et al., 2020), demonstrating the importance of vaccination route to the resulting immune response. The extended release of rMOMP in this PLGA-rMOMP formulation provided protective immunity against *C. muridarum* genital challenge and rechallenge (Sahu et al., 2021). PLGA (85:15) nanoparticle encapsulating rMOMP-187 (a peptide derivative of MOMP) induced a significant production of interleukin (IL)-6, IL-12p40, Th1/Th17 cytokines and nitric oxide (NO) by mouse J774 macrophages in a dose dependent manner and with minimal toxicity (Taha et al., 2012). In another preclinical vaccine study, spleen cells from BALB/c mice immunized with PLGA-rMOMP exhibited higher numbers of CD4^+^ and CD8^+^ T-cells and an increased secretion of IFN-γ (Th1) and IL-12p40 versus IL-4 and IL-10 (Th2) cytokines in response to restimulation with purified rMOMP, as well as immunized mice producing higher serum IgG and IgG2a (Th1) than IgG1 (Th2) rMOMP-specific antibodies (Fairley et al., 2013). The ability of PLGA-rMOMP to induce Th1 immune responses in mice makes it an extremely suitable nanoparticle-based vaccine candidate against *C. trachomatis* to be explored for translational studies.

The encapsulation of chlamydial antigens in Poly (lactic acid)-poly (ethylene glycol) or PLA-PEG, another self-adjuvating, biodegradable co-polymeric nanoparticle, has been shown to induce potent anti-*C. trachomatis* immune responses. M278, a recombinant peptide derived from *C. trachomatis* MOMP encapsulated in PLA-PEG, showed an enhancement of long-lasting chlamydia-specific CD4^+^ T-cell effector responses facilitated by caveolin-mediated endosomal antigen processing and MHC class II-dependent antigen presentation (Dixit et al., 2018). More specifically, there was an enhanced expression of pathogen-sensing receptors, such as TLR2 and Nod1, surface activation markers, including, Cd1d2 and Fcgr1, effector cytokines and chemokines, MHC class I and II molecules, as well as co-stimulatory molecules including CD40, CD80 and CD86 (Dixit et al., 2018). Subcutaneous immunization of M278 encapsulated in PLA-PEG was shown to induce secretion of IFN-γ, resulting in the differentiation of chlamydia-specific CD4^+^ T-cells to memory and effector phenotypes. Significant protection of immunized mice characterized by reduced vaginal bacterial loads after a genital tract challenge was observed and associated to elevated mucosal IgG1 and MOMP-specific IgA in the mice. Additionally, immune sera with functional neutralizing antibodies collected from mice immunized with PLA-PEG-encapsulated M278 prevented the infection of McCoy cells by *C. muridarum* (Verma et al., 2018). In another study, mice immunized with PLA-PEG encapsulated M278 generated higher T-cell cytokines [Th1 (IFN-γ, IL-2), Th17 (IL-17)] and antibodies [Th1 (IgG2a), Th2 (IgG1, IgG2b)] when compared to mice immunized with unencapsulated M278 (Dixit et al., 2014).

We recently encapsulated MOMP from *C. muridarum* (mMOMP) in the membrane-like environment of NLPs and demonstrated that vaccination with MOMP-NLPs provided a balanced T cell response with partial protection in a respiratory challenge model (He et al., 2017; Tifrea et al., 2021). We used an *Escherichia coli*-based cell-free system to express a MOMP protein from *C. muridarum* (mMOMP) and supported it within a telodendrimer nanolipoprotein particle (mMOMP–tNLP) co-localized with CpG oligodeoxynucleotide 1826 (CpG), a single-stranded synthetic DNA adjuvant, functionalized with a cholesterol tag to enable conjugation to the lipid nanoparticle. This mMOMP-tNLP complex induced an enhanced antigen-specific IgG response in vaccinated mice (He et al., 2017; Tifrea et al., 2021). We first reported a *C. muridarum*-exosomes vaccine formulated with CpG plus Montanide as adjuvants that elicited robust Th1 humoral and cell-mediated immune responses against a respiratory challenge with *C. muridarum* EB in vaccinated mice as well as producing high levels of *C. muridarum*-specific neutralizing antibodies in their serum (Pal et al., 2020).

In another study, a peptide derived from Pmp-G, a chlamydial polymorphic membrane protein, which functions as an autotransporter adhesin and plays an essential role in the initial phase of chlamydial infection (Vasilevsky et al., 2016) was encapsulated in vault nanoparticles, made from a natural nanocapsule engineered from hollow barrel-shaped eukaryotic ribonucleoprotein complexes. This vaccine formulation was shown to significantly reduce the genital bacterial burden and histopathologic inflammation in immunized mice following a *C. muridarum* challenge. Protection correlated with the induction of a systemic antigen-specific cellular immune response, characterized by an increase in PmpG-specific splenic CD4^+^ central memory T-cells and IFN-γ+ natural killer (NK) cells, a decrease in the number of inflammatory cells, such as neutrophils and TNF-α+ CD8^+^ T-cells in the genital tract, and the secretion of IFN-γ, TNF-α, IL-17, and IL-2 by splenic CD4^+^ T-cells (Jiang et al., 2017). Lastly, the self-assembly of lipid phytantriol and the immunopotentiator monomycoloyl glycerol-1 into nanocarriers with an internal hexagonal phase encapsulating chlamydial MOMP induced stronger MOMP-specific IgG humoral responses and antigen-specific CD4^+^ T-cell responses than MOMP surface-adsorbed to CAF04 liposome [a cationic liposomal adjuvant formulation containing monomycolyl glycerol [MMG] and N,N-dimethyl-N,N-dioctadecylammonium (DDA)] or unadjuvanted MOMP in immunized mice. However, CAF04 liposomes elicited more robust effector T-cell responses than the MOMP encapsulated nanocarrier, suggesting that the nanostructural composition of the lipid-based delivery system exerts a significant impact on both the type and magnitude of the induced immune responses (Rodrigues et al., 2018). Reports from the phase I clinical trial of the first in-human vaccine against *C. trachomatis* infections has shown that the use of CTH522 adjuvanted with either CAF01, a cationic liposomal adjuvant or aluminum hydroxide was safe and well tolerated (Abraham et al., 2019) (NCT02787109). While further investigation is needed, positive results from this first clinical trial showed that CTH522 formulated with CAF01 induced better humoral immunogenicity than CTH522 formulated with aluminum hydroxide (Abraham et al., 2019). These results led to another phase I clinical trial for trachoma (NCT03926728) and indicates a bright future for nanoparticle-based vaccines against *C. trachomatis*.

### 4.2 Syphilis

As with other human STIs, there is an urgent need for the development of efficacious vaccines against the spirochete *Treponema pallidum*, etiological agent for the multistage STI syphilis. Syphilis is the cause of STI-related deaths, second to only acquired immune deficiency syndrome (AIDS) (Zhao et al., 2011; Zhao et al., 2013). Syphilis exhibits alternating asymptomatic and symptomatic disease stages with different clinical manifestations. It is associated with systemic disease ranging from multiple organ impairment in adults to adverse pregnancy outcomes including abortions, premature births, stillbirths, and congenital diseases (Walker et al., 2019). Although penicillin has been an effective treatment for syphilis, its effectiveness depends on such limitations as accurate diagnosis, patient compliance, drug availability, and disease stage (Cameron and Lukehart, 2014). Hence, the need for efficacious vaccine formulations that will prevent infection and provide robust protection against all *T. pallidum* strains remains.

Despite the critical need for effective vaccines against *T. pallidum*, only a limited number of preclinical vaccine studies were conducted for decades, partly due to the fragility of the *T. pallidum* outer membrane proteins (OMPs) and limited research on *T. pallidum* biology. Current vaccine studies are investigating the role of OMPs in the induction of opsonization and phagocytosis, as well as their role as potentiators of *T. pallidum* virulence. Some of these studies utilized conventional vaccine strategies such as whole cell attenuated, aged, and γ-irradiated *T. pallidum*, while other studies have utilized *T. pallidum* specific antigens for immunization. However, only rabbits immunized with γ-irradiated *T. pallidum,* Nichols strain, over a 37-week period were shown to be fully protected against challenge with homologous treponemes but with no protection against challenge with the *T. pallidum* subspecies pallidum strain (Lithgow and Cameron, 2017). Significantly reduced bacterial organ burden in immunized rabbits after a *T. pallidum* challenge was seen in a recent study that utilized Tp0751 (a vascular adhesin of *T. pallidum* subspecies *pallidum*) as a vaccine candidate against the *T. pallidum* subspecies *pallidum* strain. The transfer of popliteal lymph nodes from Tp0751-immunized, *T. pallidum*-challenged rabbits to naive rabbits also conferred sterile protection against a *T. pallidum*-challenge (Lithgow et al., 2017). While these studies were conducted in the absence of nanoparticle formulations, these promising findings suggest that nanoparticles vaccine carriers combining these protective *T. pallidum* antigens with adjuvants could provide efficacious vaccine formulations with robust protection against all *T. pallidum* strains.

To date, the only nanoparticle-based vaccine studies against *T. pallidum* have utilized plasmids expressing IL-2 and Tp92, a *T. pallidum* OMP, encapsulated in chitosan nanoparticles individually or in combination. Both the IL-2 and Tp92 plasmid-encapsulated chitosan nanoparticle-based vaccines increased anti-Tp92 antibody levels while the combination of both plasmids encapsulated in chitosan nanoparticles showed the greatest amplification of anti-Tp92 antibodies and T-cell proliferation, which resulted in significant protection against challenge with *T. pallidum* in immunized male New Zealand white rabbits (Zhao et al., 2011). Similar effects were seen with *T. pallidum* Gpd DNA vaccine adjuvanted with IL-2 and chitosan nanoparticles (Zhao et al., 2013). These findings indicate promising vaccine outcomes from DNA complexed with nanoparticles and the need for more research on developing *T. pallidum vaccines*.

### 4.3 Gonorrhea

*Neisseria gonorrhoeae* is the bacterial agent responsible for the STI gonorrhea that poses a critical public health problem worldwide due to its resistance to multiple antimicrobials (Rowley et al., 2019). Infection with *N. gonorrhoeae* is often asymptomatic but can lead to serious complications in both men and women including urethritis, cervicitis, proctitis, pelvic inflammatory diseases, chronic pelvic pain, infertility, ectopic pregnancy, and increased susceptibility to acquiring HIV (Workowski et al., 2008; Li et al., 2013; Rice et al., 2017). The development of effective vaccine candidates against gonococcal infection is critical for the prevention and eradication of gonorrhea; however, the extraordinary ability of *N. gonorrhoeae* to change its surface antigen composition, and a lack of knowledge regarding protective correlates of immunity in humans significantly complicates vaccine development (Rotman and Seifert, 2014; Edwards et al., 2016; Gulati et al., 2019; Russell et al., 2019). Some of the gonococcal surface antigens that have been considered as vaccine targets include the porin PorB, lipo-oligosaccharide glycan structures, type IV gonococcal surface pili, and gonococcal colony opacity-associated (Opa) proteins (Russell et al., 2019).

While the nanotechnology-based preclinical studies have focused on therapeutic approaches against *N. gonorrheae* (Li et al., 2013; Lucío et al., 2020), in this study (Gala et al., 2018), the microparticles were made containing spray dried, inactivated whole-cell gonococci strain CDC-F62 loaded in biodegradable crosslinked albumin-based particulate matrix for prolonged slow antigen release. This novel nanovaccine was administered transdermally to 6–8-week-old Swiss Webster female mice using biodegradable microneedle skin patches. The immune response of vaccinated mice was monitored for 10 weeks starting immediately after the prime vaccination, followed by two booster vaccination regimens, scheduled 1 week apart. When compared to either subcutaneous or microneedle transdermal administration of *N. gonorrhoeae* antigen in suspension, the novel nanovaccine induced a higher antigen-specific IgG antibody titer from week 2 with significantly higher titers at weeks 6 and 8. At week 10, all three vaccinated groups had significantly higher levels of antigen-specific CD4^+^ and CD8^+^ T lymphocytes when compared to empty microneedles and unvaccinated mice (Gala et al., 2018). Although this whole-cell-based nanovaccine did not address the current challenge of *N. gonorrhoeae* immune evasion due to surface antigen mutations, this preclinical study opens an avenue for the possibility of *N. gonorrhoeae* infection prevention with nanoparticle-based vaccines, where multiple *N. gonorrhoeae* strains with different immunogenic epitopes can be packaged and delivered in a nanocarrier. If successful, such a nanoparticle-based vaccine will limit immune evasion by providing protection against potential *N. gonorrhoeae* strains, eliminate the strenuous processes involved in the identification of a single immunogenic epitope, and ultimately reduce the rate of acquiring multi-drug resistant infections. Hence, further exploration of the immunopotentiating capacity of nanoparticle-based vaccines for novel strategies will be essential for the development of vaccines capable of overcoming the adaptive capability of *N. gonorrhoeae*.

## 5 Recommendations for Future Nanoparticle-Based Vaccines Against Sexually Transmitted Infections

To maximize the potential of engineered nanoparticle platforms, a better understanding of the mechanisms of action of all current orthodox and non-conventional vaccine formulations and therapies that provide protection against STIs including HPV and Hepatitis B is needed. Taking advantage of the suitable properties of nanoparticles as vaccine delivery platforms that will confer sustained protection against STIs is very promising. Some of these common physiochemical properties across different types of nanoparticles that make them advantageous for vaccine delivery include their ability to conjugate, encapsulate or adsorb vaccine molecules; their size and surface area, which determines the mode of cellular uptake and specificity; their hydrophobicity, which plays an integral role in interaction with immune cells and soluble proteins; their surface charge, which can be modified for targeted vaccine delivery; and their shape, which is critical for cellular interaction, intracellular trafficking and antigen release rate (Gatoo et al., 2014; Pati et al., 2018). In this review, we discussed the different nanoparticle-based vaccine platforms and their immunological benefits including improved access to lymphatic system, optimal packaging and presentation of antigens, and better induction of immune responses (Singh, 2021). In general, only a few nanoparticle-based vaccines have been used in advanced clinical trials for infectious diseases including influenza (NCT03293498, NCT03658629) and respiratory syncytial virus (Stephens and Varga, 2020) (NCT01960686, NCT02247726 and NCT02624947), and recently for Epstein-Barr virus (NCT04645147) and COVID-19 (NCT05007951). Although a handful of these platforms have been implemented for bacterial STI preclinical vaccine research, there is a critical need for more research and development that will translate these formulations to the clinics for all three STIs discussed in this review.
